# Prevalence of prehypertension and associated risk factors among Chinese adults from a large-scale multi-ethnic population survey

**DOI:** 10.1186/s12889-016-3411-4

**Published:** 2016-08-11

**Authors:** Tao Xu, Junting Liu, Guangjin Zhu, Junxiu Liu, Shaomei Han

**Affiliations:** 1 Institute of Basic Medical Sciences, Chinese Academy of Medical Sciences & School of Basic Medicine, Peking Union Medical College, Beijing, 100005 China; 2 Department of Epidemiology, Capital Institute of Pediatrics, Beijing, 100020 China; 3 Department of Epidemiology and Biostatistics, University of South Carolina, United States, Columbia, SC 29208 USA

**Keywords:** Prehypertension, China, Adult, Risk factor, Ethnicity

## Abstract

**Background:**

Up to date, most of previous studies about Chinese prehypertension were conducted based on a small sample or in only one province, which could not represent the general population in China. Furthermore, no information on the ethnic difference in prevalence of prehypertension has been reported in China. The aim of this study is to examine the sex-specific, age-specific and ethnic-specific prevalence of prehypertension and associated risk factors in a large-scale multi-ethnic Chinese adult population.

**Methods:**

The subjects came from a large-scale population survey about Chinese physiological constants and health conditions conducted in six provinces. 47, 495 adults completed blood pressure measurement. Prehypertension was defined as not being on antihypertensive medications and having SBP of 120–139 mmHg and/or DBP of 80–89 mmHg. Odds ratio (OR) and its 95 % confidence interval (CI) from logistic models were used to reflect the prevalence of prehypertension.

**Results:**

The mean age of all subjects was 43.9 ± 16.8 years. The prevalence of hypertension and prehypertension for all them was 29.5 and 36.4 %, respectively. The prevalence of hypertension and prehypertension for males (33.2 and 41.1 %, respectively) was higher than that for females (27.0 and 33.2 %, respectively), and *P* < 0.001. The mean age of the subjects was 54.8 ± 14.0 years for hypertensive, 44.0 ± 16.0 years for prehypertensive and 35.3 ± 14.5 years for normotensive. With aging, subjects had more odds of getting prehypertension. Multivariate logistic model indicated that males (OR = 2.076, 95 % CI: 1.952–2.208), laborers with mental work (OR = 1.084, 95 % CI: 1.020–1.152), Yi (OR = 1.347, 95 %CI: 1.210–1.500) and Hui subjects (OR = 1.133, 95 % CI: 1.024–1.253), alcohol drinkers (OR = 1.147, 95 % CI: 1.072–1.228), the generally obese (OR = 2.460, 95 % CI: 2.190–2.763), the overweight (OR = 1.667, 95 % CI: 1.563–1.788), the abdominally obese (OR = 1.371, 95 % CI: 1.280–1.467) and subjects with family history of cardiovascular diseases (OR = 1.132, 95 % CI: 1.045–1.226) had higher prevalence of prehypertension. Subjects with higher educational level (OR = 0.687, 95 % CI: 0.627–0.752 for university) and Miao (OR = 0.753, 95 % CI: 0.623–0.910), Tibetan (OR = 0.521, 95 % CI: 0.442–0.613), Tujia (OR = 0.779, 95 % CI: 0.677–0.898) subjects had lower prevalence.

**Conclusion:**

High prevalence rate of prehypertension was general in Chinese adults. Many sociodemographic characteristics were significantly associated with prehypertension. There were important clinical significance and public health significance about age-specific, gender-specific and ethnic-specific Chinese prehypertension conditions studies.

## Background

Cardiovascular diseases (CVD) and cerebrovascular diseases have gradually become the leading cause of mortality in both developed and developing countries over the last several decades. CVD has become the No. 1 cause of death 10 years ago in China [1]. Although CVD and cerebrovascular diseases were caused by many risk factors, hypertension has been identified as the major one leading to myocardial infarction, stroke, renal dysfunction and heart failure [2, 3]. In general, the trend of hypertension prevalence has been increasing in many countries. It was reported that hypertension affected 26.4 % of the world’s adult population (972 million) in 2000, and the rates were expected to increase to 29.2 % (1.56 billion) by 2025 [4]. China has the most condensed population in the world and has also been transitioning economically. As a major public health problem, hypertension is threatening more and more Chinese people too. In China, hypertension has become a dominant chronic disease and appropriately 160 million Chinese or 18.8 % were suffering from hypertension according to the recent report from the Chinese National Nutrition and Health Survey [5].

The Eighth Joint National Committee on the Prevention, Detection, Evaluation and Treatment of High Blood Pressure (JNC-8) continued to recommend and emphasize lifestyle changing in prevention of hypertension [6, 7]. In 2014, JNC-8 introduced a new category of prehypertension which is defined as systolic blood pressure (SBP) from 120 to 139 mmHg, and/or diastolic blood pressure (DBP) from 80–89 mmHg in order to improve blood pressure control [6, 7]. After the release of this new blood pressure category, numerous studies have been focusing on this topic. Research on prehypertension prevalence and its associated risk factors is of great interest worldwide [8, 9]. More than 30 % of adults were reported suffering from prehypertension in the United States [10]. It was estimated that prehypertension progresses to clinical hypertension at a rate of 19 % over 4 years [11]. According to the Framingham Heart Study, people with prehypertension are 3.5 times more likely to develop heat attacks than those with normal blood pressure [12].

However, little is known about the current situation of the prevalence of prehypertension in China. Most of previous estimates on the prevalence of prehypertension in china were only based on only one province of China or a small sample size, which could not represent the general Chinese population [13–16]. Furthermore, China is a multi-ethnic country, up to date no information on the ethnic difference in prevalence of prehypertension has been reported in China. To the best of our knowledge, this is the first time to report the prevalence of prehypertension and its associated risk factors among Chinese adult population using nationally representative sample covering dozens of minorities. Specifically, the aim of this study is to examine the sex-specific, age-specific and ethnic-specific prevalence of hypertension and prehypertension and associated risk factors in a large-scale multi-ethnic Chinese adult population.

## Methods

### Sample and participants

This study is based on data from a large-scale population survey in a representative sample of general Chinese population from 2007 to 2011. The main purpose of that survey is to examine Chinese physiological constants and health conditions. It was supported by the basic performance key project by the ministry of science and technology of the People’s Republic of China, and has been approved by the review board of Institute of Basic Medical Sciences, Chinese Academy of Medical Sciences annually. In brief, this survey was carried out in six provinces of China, which was selected according to the population size of the minorities, including Sichuan province, Heilongjiang province, Hunan province, Inner Mongolia autonomous region, Yunnan province and Ningxia Hui autonomous region. Three-stage cluster sampling method was used to select eligible subjects in each province. First, two or three cities were sampled according to their economical status and ethnic inhabitations of minorities. Then five or six communities and villages were randomly selected in each city. Among eligible people, we excluded participants suffering from severe chronic diseases and those with a high fever in the past 15 days. All included participants gave their informed consent to participant in the study. The procedure of this study involves physical examination including blood pressure measurement and face-to-face questionnaire interviews. Finally, out of 52, 265 included subjects, 47, 495 (90.9 %) subjects have complete data on all survey scales and blood pressure measure.

### Blood pressure measurement

Blood pressure was measured by trained physicians following standard guidelines, which involved American national high blood pressure education program and Chinese guidelines for hypertension prevention and treatment [17, 18]. Subjects were asked to avoid drinking coffee, tea or eating other stimulant food and drugs before the measurement. Blood pressure was measured at morning after subjects having rested for five minutes in the seating position with her or his back supported, feet on the floor and right arm supported with cubit fossa at heart level. The appropriate cuff was chosen based on their arm circumference. Blood pressure was measured based on one clinical visit.

Average values of two readings separated by 5 min were used in the analysis. The classification of normotensive (NT), prehypertensive (PRT) and hypertensive (HT) was based on the criteria from JNC-8 [6, 7]. Subjects were defined as being hypertensive if they had an average SBP equal to or greater than 140 mmHg, and/or average DBP equal to or greater than 90 mmHg, and/or were diagnosed as being hypertensive in the past, and/or reported current treatment with antihypertensive medications. Prehypertension was defined as not being on antihypertensive medications and having SBP of 120–139 mmHg and /or DBP of 80–89 mmHg. Normal blood pressure was defined as not being on antihypertensive medications and having SBP <120 mmHg and DBP <80 mmHg.

### Sociodemographic variables

Sociocharacteristics included age, gender, marital status, educational level, occupation, ethnicity, smoking, alcohol drinking, high-salt diet condition, body mass index (BMI), abdominal obesity and family history of cardiovascular diseases. Age was categorized into 6 groups: 18–29, 30–39, 40–49, 50–59, 60–69 and 70–79 years old. Marital status included married, single, divorced and widowed. Educational level was categorized into three groups: illiterate or primary school, high school and university. Occupation referred to physical laborer or laborer with mental work. With broad range of ethnicity, subjects were grouped as Han, Yi, Miao, Mongolia, Tibetan, Hui, Tujia, Korean, Manchu, Yao, Dai, Qiang and all others. Smoking was categorized into never smokers, current smokers and former smokers. Alcohol drinking was categorized into regular drinkers and non-drinkers. Subjects were also categorized as liking high-salt diet or not. BMI was defined as weight (kg) divided by squared height (m^2^). Weight and height were measured to the nearest 0.1 kg or 0.1 cm with the subjects wearing light undergarments and no shoes according to standard procedure [19, 20]. According to Chinese guidelines for prevention and control of adult overweight and obesity and the Chinese criteria by Working Group on Obesity in China [19, 20], general obesity is defined as BMI equal to or greater than 28 kg/m^2^; overweight was defined as BMI less than 28 kg/m^2^ and equal to or greater than 24 kg/m^2^. Waist circumference (WC) was measured to the nearest 0.1 cm while subjects were in the standing position. According to the Chinese criteria by Working Group on Obesity in China [20], abdominal obesity is defined as WC equal to or greater than 85 cm for males and 80 cm for females. Finally, in order to account for the genetic variation, subjects were categorized into having family history of cardiovascular diseases or not. The subject was defined having family history of cardiovascular diseases if his/her father or/and mother have been diagnosed suffering from cardiovascular diseases.

### Statistical analysis

Database was constructed with EPI3.02 software by two data managers respectively and was corrected to guarantee the accuracy and integration of the data. Continuous variables were described with mean and standard deviation (SD). Categorical variables were described with number and percentage. The mean age was compared with analysis of variance. Blood pressure conditions were compared with Wilcoxon rank sum test. Univariate and multivariate logistic regression model was used to estimate odds ratio (OR) and its 95 % confidence interval (CI) for prehypertension. Variables with *P* < 0.05 in univariate analyses were included in the multivariate logistic model. All statistical analysis was conducted in SAS9.2 software (SAS institute Inc, Cary, NC, USA). Two sided *P* < 0.05 was considered statistically significant.

## Results

Of all subjects, 16,211 individuals had normal blood pressure, 17,281 individuals were prehypertensive and 14,003 were hypertensive. The prevalence rates of HT and PRT were 29.5 and 36.4 % respectively. The mean age for NT subjects was 35.3 ± 14.5 years old, which seems younger than those with HT (54.8 ± 14.0) or PRT (44.0 ± 16.0), *P* < 0.001.

The basic characteristics were tabulated according to three blood pressure categories in Table 1. Subjects who are older (*P* < 0.001), male (*P* < 0.001), not single (*P* < 0.001), physical laborer (*P* < 0.001), lower educational level (*P* < 0.001), Yi and Korean (*P* < 0.001), smokers (*P* < 0.001), drinkers (*P* < 0.001), liking high-salt diet (*P* = 0.003), general obese or overweight (*P* < 0.001), abdominal obese (*P* < 0.001) and with family history of cardiovascular diseases (*P* < 0.001) have higher prevalence of HRT. The prevalence rates of HT and PRT were 33.2 and 41.1 % for males, 27.0 and 33.2 % for females respectively. 40.8 % of subjects in 18–29 years old were HT or PRT, but 89.7 % of subjects in seventies were HT or PRT. With aging, the proportion of NT subjects declined gradually. The prevalences of HT and PRT across demographic variables were shown in Table 1.

**Table 1:** Prevalence conditions of hypertension and prehypertension (n(%))

Characteristics	HT (*n* = 14003)	PRT (*n* = 17281)	NT (*n* = 16211)	*Z/χ* ^2 ^^*^	*P*
Gender				27.591	<0.001
Male	6378 (33.2)	7902 (41.1)	4950 (25.7)		
Female	7625 (27.0)	9379 (33.2)	11261 (39.8)		
Age				9718.166	<0.001
18–29	729 (6.1)	4303 (34.7)	7007 (58.2)		
30–39	1356 (17.0)	3145 (39.4)	3483 (43.6)		
40–49	2840 (29.4)	3906 (40.4)	2930 (30.3)		
50–59	3525 (43.1)	3031 (37.1)	1621 (19.8)		
60–69	3455 (55.7)	1930 (31.1)	817 (13.2)		
70–79	2098 (61.4)	966 (28.3)	353 (10.3)		
Marital status				4733.453	<0.001
Married	12051 (34.7)	12878 (37.1)	9792 (28.2)		
Single	645 (6.4)	3595 (35.4)	5902 (58.2)		
Divorced/widowed	1307 (49.7)	808 (30.7)	517 (19.6)		
Educational level				3391.819	<0.001
Illiterate or primary school	4505 (46.5)	3225 (33.3)	1968 (20.3)		
High school	6906 (31.8)	8239 (37.9)	6591 (30.3)		
University	2592 (16.1)	5817 (36.2)	7652 (47.6)		
Occupation				49.867	<0.001
Physical laborer	9684 (38.7)	8820 (35.3)	6512 (26.0)		
Laborer with mental work	4319 (19.2)	8461 (37.6)	9699 (43.1)		
Ethnicity				332.522	<0.001
Han	10789 (30.3)	12874 (36.2)	11927 (33.5)		
Yi	652 (26.7)	985 (40.3)	808 (33.0)		
Miao	143 (22.2)	250 (38.9)	250 (38.9)		
Mongolia	557 (31.6)	644 (36.6)	560 (31.8)		
Tibetan	138 (15.6)	275 (31.1)	471 (53.3)		
Korean	529 (36.5)	525 (36.2)	395 (27.3)		
Hui	732 (28.2)	968 (37.3)	896 (34.5)		
Tujia	293 (24.2)	444 (36.6)	475 (39.2)		
Others	170 (18.6)	316 (34.5)	429 (46.9)		
Smoker				374.036	<0.001
Never	10396 (28.1)	13092 (35.4)	13473 (36.5)		
Current	3515 (34.0)	4133 (39.9)	2701 (26.1)		
Former	92 (49.7)	56 (30.3)	37 (20.0)		
Alcohol drinker				19.417	<0.001
No	10360 (28.1)	12992 (35.3)	13466 (36.6)		
Yes	3643 (34.1)	4289 (40.2)	2745 (25.7)		
High-salt diets				2.962	0.003
No	12479 (29.3)	15522 (36.4)	14617 (34.3)		
Yes	1524 (31.2)	1759 (36.1)	1594 (32.7)		
BMI				6399.594	<0.001
General obesity	3316 (58.8)	1776 (31.5)	551 (9.8)		
Overweight	5886 (40.1)	5763 (39.3)	3026 (20.6)		
Normal	4801 (17.7)	9742 (35.8)	12634 (46.5)		
Abdominal obesity				76.4597	<0.001
Yes	8691 (47.0)	6473 (36.4)	3074 (16.6)		
No	5312 (18.3)	10538 (36.4)	13137 (45.3)		
Family history of cardiovascular diseases				8.0453	<0.001
No	12385 (29.0)	15533 (36.4)	14791 (34.6)		
Yes	1618 (33.8)	1748 (36.5)	1420 (29.7)		

*Wilcoxon rank-sum test was used, in which the statistic was Z or *χ* ^*2*^

*HT* hypertension *PRT* prehypertension, *NT* Normotension

Table 2 gives the results of the univariate and multivariate logistic regression analysis of risk factors for prehypertension. After controlling other demographic characteristics, males had more than twice odds of getting prehypertension (OR = 2.076, 95 % CI: 1.952–2.208) than females. Subjects had more odds of getting prehypertension with aging. In univariate analysis, subjects who are single had lower odds of getting prehypertension while the divorced or widowed was in opposite direction. However, marital status was not statistically associated with prehypertension after controlling other demographic characteristics. In two logistic models, educational level was closely associated with prehypertension and higher educational level could reduce the prevalence compared to illiterate subjects or ones with primary school educational level. Compared to Han subjects, Yi and Hui subjects had a higher prevalence of prehypertension, but Miao, Tibetan and Tujia subjects were associated with a lower prevalence rate of prehypertension. Contrary to univariate analysis results, current smokers had lower prevalence hazard (OR = 0.741, 95 % CI: 0.688–0.798) in the multivariate model. However association between former smoker and prehypertension was not statistically significant. Alcohol drinking (OR = 1.147 95 % CI: 1.072–1.228) and family history of cardiovascular diseases (OR = 1.132, 95 % CI: 1.045–1.226) had similar effect on the prevalence of prehypertension. Abdominal obesity, general obesity or overweight could increase prevalence of prehypertension. In summary, after controlling other demographic characteristics, gender, age, educational level, occupation, ethnicity, smoking, alcohol drinking, BMI, abdominal obesity and family history of cardiovascular diseases were associated with prehypertension.

**Table 2:** Risk factors associated with prehypertension

Characteristics	Univariate logistic model		Multivariate logistic model	
	OR^*^	95 % CI^*^	OR^*^	95 % CI^*^
Gender				
Male	1.917	1.833–2.005	2.076	1.952–2.208
Female	1.0 (Ref.)		1.0 (Ref.)	
Age				
18–29	1.0 (Ref.)		1.0 (Ref.)	
30–39	1.470	1.383–1.563	1.201	1.090–1.322
40–49	2.171	2.042–2.308	1.607	1.456–1.773
50–59	3.045	2.835–3.270	2.081	1.868–2.319
60–69	3.847	3.515–4.210	2.498	2.204–2.831
70–79	4.456	3.922–5.063	2.948	2.514–3.457
Marital status				
Married	1.0 (Ref.)		1.0 (Ref.)	
Single	0.463	0.441–0.486	0.928	0.844–1.020
Divorced/widowed	1.188	1.061–1.331	1.003	0.888–1.134
Educational level				
Illiterate or primary school	1.0 (Ref.)		1.0 (Ref.)	
High school	0.763	0.715–0.814	0.919	0.851–0.992
University	0.464	0.434–0.495	0.687	0.627–0.752
Occupation				
Physical laborer	1.0 (Ref.)		1.0 (Ref.)	
Laborer with mental work	0.644	0.617–0.673	1.084	1.020–1.152
Ethnicity				
Han	1.0 (Ref.)		1.0 (Ref.)	
Yi	1.129	1.026–1.244	1.347	1.210–1.500
Miao	0.926	0.776–1.106	0.753	0.623–0.910
Mongolia	1.065	0.949–1.196	1.042	0.920–1.179
Tibetan	0.541	0.465–0.629	0.521	0.442–0.613
Korean	1.231	1.078–1.406	0.965	0.837–1.111
Hui	1.001	0.911–1.100	1.133	1.024–1.253
Tujia	0.866	0.759–0.988	0.779	0.677–0.898
Others	0.682	0.589–0.791	0.974	0.832–1.140
Smoker				
Never	1.0 (Ref.)		1.0 (Ref.)	
Current	1.575	1.492–1.662	0.741	0.688–0.798
Former	1.558	1.028–2.361	0.825	0.530–1.285
Alcohol drinker				
No	1.0 (Ref.)		1.0 (Ref.)	
Yes	1.619	1.535–1.709	1.147	1.072–1.228
High-salt diets				
No	1.0 (Ref.)		1.0 (Ref.)	
Yes	1.039	0.968–1.116	0.977	0.905–1.055
BMI				
General obesity	4.180	3.785	2.460	2.190–2.763
Overweight	2.470	2.346	1.667	1.563–1.788
Normal	1.0 (Ref.)		1.0 (Ref.)	
Abdominal obesity				
Yes	2.734	2.601–2.873	1.371	1.280–1.467
No	1.0 (Ref.)		1.0 (Ref.)	
Family history of cardiovascular diseases				
No	1.0 (Ref.)		1.0 (Ref.)	
Yes	1.172	1.089–1.262	1.132	1.045–1.226

*OR odds ratio, *CI* confidence interval

## Discussion

In developed counties and developing counties, prevalence of hypertension and prehypertension was different from others. In general, the prevalence of hypertension has been highest in industrial countries. For example, Wang Y mentioned that approximately 60 % of American adults have prehypertension or hypertension [10]. In China, the overall prehypertension prevalence rate was estimated to be 21.9 % among Chinese people aged from 35–74 years old from the InterASIA study conducted 10 years ago [21]. Compared to InterASIA study results, the prevalence rate of prehypertension were found increased greatly in the present study, in which the prevalence rates of hypertension and prehypertension were 29.5 and 36.4 % respectively in China. All previous hypertension survey about Chinese people were conducted in a province of China and didn’t include Chinese minorities [13, 14, 21], which was not representative for the general population in China. The present study is the first study to investigate the prevalence of prehypertension in a representative sample of general Chinese population with the largest sample size covering dozens of ethnicities. Given that our analysis excluded subjects suffering from some severe chronic diseases and having fever in the last 15 days, we believe the true prevalence of hypertension and prehypertension in general Chinese adults would be underestimated. This is an important public health issue and more attention should be paid to provide health education for hypertensive and prehypertensive people to treat their hypertension regularly.

Multivariate logistic regression model analysis found that gender, age, educational level, occupation, ethnicity, smoking, alcohol drinking, BMI, abdominal obesity and family history of cardiovascular diseases were associated with prehypertension. Male subjects had twice risk of prehypertenion and then males should pay more attention to blood pressure control. Higher educational level was a protective factor of prehypertension, which was similar as the findings from industrialized countries, in which high educational level was found to be associated with lower risk of hypertension [22–24]. People with higher educational level maybe have learned more health knowledge about blood pressure and developed more healthy life styles. Increased knowledge of a healthy life-style in order to prevent hypertension is of importance to public health. China is a multi-ethnic country and minorities have their specific dietary habits and life styles, which could influence their blood pressure status. We found that prevalence of prehypertension were statistically different between ethnicities. Both Okosun IS et al. and Agyemang C et al. have ever found that ethnic difference can influence the prevalence of hypertension and prehypertension [25, 26]. Subjects with family history of cardiovascular diseases had higher risk of prehypertension like previous findings [27, 28]. Consequently, people whose parents have suffered from cardiovascular diseases should supervise their blood pressure level more frequently. In contrary to univariate analysis, marital status was not statistically significant in multivariate logistic analysis. The possible cause was that the single was younger than others, which would be verified insignificant after adjusting age in multivariate analysis.

Life style factors were found closely associated with prehypertension. Contrary to the results in univariate analysis, current smokers had lower prevalence for prehypertension. Although association of smoking and prehypertension needs to be explored further, other studies reported similar results as this paper [13, 18, 29]. In this study, alcohol drinking was found as a risk factor for prehypertension. Fan AZ has ever reported that there was a direct association between higher alcohol consumption and a higher prevalence rate of prehypertension among non-hypertensive drinkers [30]. After controlling other demographic characteristics, general obesity had nearly 2.5-fold prevalence and overweight had nearly twice prevalence of prehypertension. Significant positive associations between overweight, general obesity, abdominal obesity and prehypertension were same as reported in previous reports [13, 31–33]. So, reducing alcohol drinking and keeping in shape would facilitate the control of blood pressure. Furthermore, although some reports have mentioned high-salt diet could increase risk for hypertension [34, 35], association of high-salted diet and prehypertension was not statistically significant in the present paper. This may be caused by recall bias resulted from self-reported diet conditions.

Several limitations have to be mentioned. First, due to the nature of cross-sectional study design, we could not make causation between potential risk factors and prehypertension. Second, criteria of defining prehypertension and hypertension only based on one time measure of blood pressure without follow-up information might not truly represent the prehypertension and hypertension status. Hypertension could of course be defined in different ways, one way is self-reported, and another way is that it is based on diagnosis in patient records or administrative databases. The different definitions of prehypertension and hypertension limit the comparison of prevalence figures from different studies. Finally, recall bias might exist in the current study due to self-report high-salt diet conditions.

## Conclusions

Appropriately two-third of subjects was hypertensive or prehypertensive and high prevalence rate of prehypertension was general in Chinese adults. Many sociodemographic characteristics were significantly associated with hypertension and prehypertension conditions. There were important clinical significance and public health significance about age-specific, gender-specific and ethnic-specific hypertension and prehypertension studies in China. More health education should be provided for hypertensive people to treat their hypertension regularly. In addition, regular medical check-up and measuring blood pressure are necessary to improve awareness, treatment and control of hypertension.

## Abbreviations

BMI, body mass index; CI, confidence interval; DBP, diastolic blood pressure; HT, hypertensive; JNC-8, the Eighth Joint National Committee on the Prevention, Detection, Evaluation and Treatment of High Blood Pressure; NT, normotensive; OR, Odds ratio; PRT, prehypertensive; SBP, systolic blood pressure; SD, standard deviation; WC, Waist circumference; WGOC, Working Group on Obesity in China
